# Book Review: The Elephant in the Brain: Hidden Motives in Everyday Life

**DOI:** 10.3389/fpsyg.2021.668717

**Published:** 2021-03-30

**Authors:** Jing Zhang

**Affiliations:** ^1^Institute of Psychological Health, Hangzhou Dianzi University, Hangzhou, China; ^2^School of Medicine, Technische Universität Dresden, Dresden, Germany

**Keywords:** the elephant in the brain, hidden motives, selfishness, self-deception, subconsciousness, social behavior

Whether animals have consciousness is still under debate, but we agree that human beings do have. We perform various tasks when we are awake, and it seems there are conscious motives behind almost every intentional activity. Freud's work makes us apprehend that subconsciousness can sometimes substantially impact our actions and decisions; it does not prevent us from giving multiple conscious motivational explanations for our expressions and actions in social life. Therefore, if someone tells us that our motive is not what we say or what we know, it must be challenging to accept (Gazzaniga, 2011). Nevertheless, is it possible that this is the case? In the book *The Elephant in the Brain: Hidden Motives in Everyday Life*, Kevin Simler and Robin Hanson aspire to demonstrate that there may be varied hidden motives that we are unaware of behind our social behaviors.

The book's main title comes from an English saying, “the elephant in the room,” which describes the phenomenon that people deliberately avoid and ignore an apparent problem. Using “the elephant in the brain,” they try to express that we turn a blind eye to something that exists in our brain.

Part I, “Why We Hide Our Motives,” aims to show that there are various motives behind our actions, but we tend to focus on and exaggerate good, prosocial motives while downplaying ugly and selfish ones. The authors start from animals' social behavior (Chapter 1, “Animal Behavior”). The fact that primates spend much more time than necessary (meet the health requirement) suggests that social grooming may have other functions, such as building trust with each other through mutual grooming and forming alliances that can help them in other situations (Dunbar, 2010). Further, the authors mention competitive altruism that some animals compete for providing food and protection for their groupmates. By citing these examples, Simler and Hanson want to explicate that even the motives behind animals' behavior may be complicated; there are often deeper motives behind human (as higher primates) behaviors.

Chapter 2 (“Competition”) continues the evolutionary perspective. Human beings' behaviors are formed and maintained out of evolutionary considerations, and competition is inevitable in evolution. The three main types of competition for our ancestors were probably sex, social status, and politics. No matter which game field the competition took place, the intra-species competition would often waste resources. Human beings have formed norms in order to limit waste. Chapter 3 (“Norms”) combs the history of norm formation and explains why we need norms and how gossip and reputation play a role in forming and maintaining norms. In theory, a successful norm-enforcement can reduce the use of the brain. However, the fact is that human brains are getting bigger instead of shrinking. A larger brain consumes more energy; therefore, it must be decisive for human survival—one of the influence factors may be the existence of cheating.

In Chapter 4 (“Cheating”), the authors exclaim that “Everybody cheats.” The mutual restriction between norm-enforcers and norm-evaders improves their mental abilities (Trivers, 2011). There are different forms of cheating: “cheating on a test” is a norm-evasion scenario that whether a particular person (the professor) has detected, while “drinking in public” is a scenario that how many people know. Also, there is a special cheating—self-deception. Concerning the function of self-deception, the authors introduce two hypotheses (Chapter 5, “Self-deception”). One recognizes self-deception as a way of self-defense. The other considers self-deception as an outward-facing, manipulative, and ultimately self-serving mechanism rather than the inward-facing, defensive, self-defeating mechanism suggested by Freud. The authors agree more with the latter. We show self-deception in multiple situations, of which motivational self-deception is essential (Chapter 6, “Counterfeit Reasons”). We strategically ignore our motives. In other words, we do not always know what is behind our actions, but we pretend to know. In most cases, we even cannot notice that we are just pretending to know.

In a nutshell, in Part I, the authors lead us to face the elephant in our brain and demonstrate how it came into being by quoting research and evidence from microsociology, psychology, primatology, and economics. “The elephant in the brain” refers to human beings' selfishness and a collection of related concepts or expressions. Then Part II, “Hidden Motives in Everyday Life,” further illustrates the elephant's widespread existence and its far-reaching impact on our lives through concrete examples.

There are 10 cases in Part II (“Body Language,” “Laughter,” “Conversation,” “Consumption,” “Art,” “Charity,” “Education,” “Medicine,” “Religion,” and “Politics”), which cover diverse aspects of our social life. The authors introduce evident motives for each one, point out that they are insufficient to explain, and finally give alternative motives. All the chapters in Part II are independent of each other; readers can skip according to interests without undermining comprehension. Besides demonstrating the universality and importance of hidden motives, the authors emphasize that the ultimate goal of confronting the elephant in our brain is using our acknowledgment to behave better, rather than justifying selfishness. This book's significant highlight is to test that “there are hidden motives behind human behaviors” in wide-ranging aspects. Nonetheless, the authors cannot avoid choosing evidence according to their argument's needs. For example, spending money on others can make people happier than those who spend it on themselves, regardless of how much money or whether a third party knows it or not (Dunn et al., 2008). Hidden motives cannot satisfactorily explain such human behavior. In any case, the book is still worth reading, whether for amateur or professional practitioners of sociology, psychology, economics, political science, or other related disciplines.

## Author Contributions

The author confirms being the sole contributor of this work and has approved it for publication.

## Conflict of Interest

The author declares that the research was conducted in the absence of any commercial or financial relationships that could be construed as a potential conflict of interest.
